# 1-Benzyl-3-[(dimethyl­amino)methyl­ene]-4-phenyl-1*H*-1,4-benzodiazepin-2(3*H*)-one

**DOI:** 10.1107/S1600536809031687

**Published:** 2009-08-15

**Authors:** Redwan Mohamed Zemama, Ibtissam Amari, Rachid Bouhfid, El Mokhtar Essassi, Seik Weng Ng

**Affiliations:** a Laboratoire de Chimie Organique Hétérocyclique, Pôle de Compétences Pharmacochimie, Université Mohammed V-Agdal, BP 1014 Avenue Ibn Batout, Rabat, Morocco; b Institute of Nanomaterials and Nanotechnology, Avenue de l’Armée Royale, Madinat El Irfane, 10100 Rabat, Morocco; c Department of Chemistry, University of Malaya, 50603 Kuala Lumpur, Malaysia

## Abstract

The title compound, C_25_H_23_N_3_O, features a benzene ring fused with a seven-membered 1,4-diazepine ring; the latter ring adopts a boat conformation with the (dimethyl­amino)methyl-bearing C atom as the prow and the fused-ring C atoms as the stern. There are two independent mol­ecules in the asymmetric unit with similar conformations.

## Related literature

1,5-Benzodiazepines are synthons for other heterocyclic compounds; for the crystal structure of related 1,5-benzodiazepines, see: Doubia *et al.* (2007*a* ▶,*b* ▶).
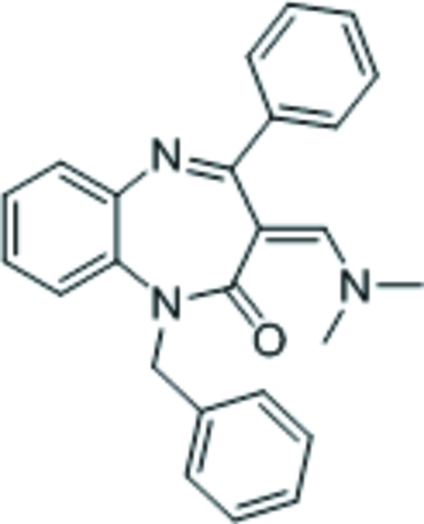


## Experimental

### 

#### Crystal data

C_25_H_23_N_3_O
*M* *_r_* = 381.46
Monoclinic, 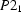

*a* = 8.9733 (3) Å
*b* = 26.3103 (7) Å
*c* = 9.9312 (3) Å
β = 115.644 (1)°
*V* = 2113.7 (1) Å^3^
*Z* = 4
Mo *K*α radiation
μ = 0.07 mm^−1^
*T* = 293 K
0.3 × 0.3 × 0.3 mm

#### Data collection

Bruker APEX2 diffractometer
Absorption correction: none
25753 measured reflections
4959 independent reflections
4091 reflections with *I* > 2σ(*I*)
*R*_int_ = 0.038

#### Refinement

*R*[*F* ^2^ > 2σ(*F* ^2^)] = 0.043
*wR*(*F* ^2^) = 0.118
*S* = 1.03
4959 reflections
527 parameters
1 restraint
H-atom parameters constrained
Δρ_max_ = 0.25 e Å^−3^
Δρ_min_ = −0.17 e Å^−3^

### 

Data collection: *APEX2* (Bruker, 2005 ▶); cell refinement: *SAINT* (Bruker, 2005 ▶); data reduction: *SAINT*; program(s) used to solve structure: *SHELXS97* (Sheldrick, 2008 ▶); program(s) used to refine structure: *SHELXL97* (Sheldrick, 2008 ▶); molecular graphics: *X-SEED* (Barbour, 2001 ▶); software used to prepare material for publication: *publCIF* (Westrip, 2009 ▶).

## Supplementary Material

Crystal structure: contains datablocks global, I. DOI: 10.1107/S1600536809031687/tk2525sup1.cif

Structure factors: contains datablocks I. DOI: 10.1107/S1600536809031687/tk2525Isup2.hkl

Additional supplementary materials:  crystallographic information; 3D view; checkCIF report

## Figures and Tables

**Fig. 1.**
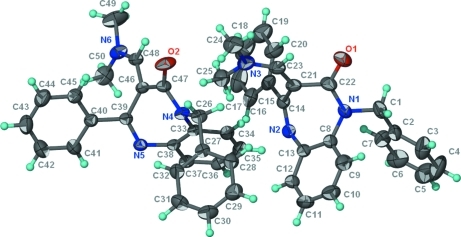
Thermal ellipsoid plot (Barbour, 2001) of the two independent molecules of C25H23N3O at the 50% probability level; hydrogen atoms are drawn as spheres of arbitrary radius.

## supplementary crystallographic information

### Experimental

A mixture of 1-benzyl-4-phenyl-1,5-benzodiazepin-2-one (1 g, 3.06 mmol) and dimethylformamide-dimethylacetal (1.23 ml, 9.18 mmol) was heated at 413 K for 4 h. The product was recrystallized from ethanol to give yellow crystals.

### Refinement

Carbon-bound H-atoms were placed in calculated positions (C—H 0.93 to 0.97 Å) and were included in the refinement in the riding model approximation with *U*(H) set to 1.2 to 1.5*U*_eq_(C).

In the absence of significant anomalous scattering effects, 4583 Friedel pairs were averaged in the final refinement.

### Crystal data

**Table:**

C_25_H_23_N_3_O	*F*(000) = 808
*M**_r_* = 381.46	*D*_x_ = 1.199 Mg m^−^^3^
Monoclinic, *P*2_1_	Mo *K*α radiation, λ = 0.71073 Å
Hall symbol: P 2yb	Cell parameters from 9099 reflections
*a* = 8.9733 (3) Å	θ = 2.3–28.2°
*b* = 26.3103 (7) Å	µ = 0.07 mm^−^^1^
*c* = 9.9312 (3) Å	*T* = 293 K
β = 115.644 (1)°	Block, yellow
*V* = 2113.7 (1) Å^3^	0.3 × 0.3 × 0.3 mm
*Z* = 4	

### Data collection

**Table:**

Bruker APEX2 diffractometer	4091 reflections with *I* > 2σ(*I*)
Radiation source: fine-focus sealed tube	*R*_int_ = 0.038
graphite	θ_max_ = 27.5°, θ_min_ = 2.3°
φ and ω scans	*h* = −11→11
25753 measured reflections	*k* = −33→34
4959 independent reflections	*l* = −12→12

### Refinement

**Table:**

Refinement on *F*^2^	Primary atom site location: structure-invariant direct methods
Least-squares matrix: full	Secondary atom site location: difference Fourier map
*R*[*F*^2^ > 2σ(*F*^2^)] = 0.043	Hydrogen site location: inferred from neighbouring sites
*wR*(*F*^2^) = 0.118	H-atom parameters constrained
*S* = 1.03	*w* = 1/[σ^2^(*F*_o_^2^) + (0.0708*P*)^2^ + 0.1173*P*] where *P* = (*F*_o_^2^ + 2*F*_c_^2^)/3
4959 reflections	(Δ/σ)_max_ = 0.001
527 parameters	Δρ_max_ = 0.25 e Å^−^^3^
1 restraint	Δρ_min_ = −0.17 e Å^−^^3^

### Fractional atomic coordinates and isotropic or equivalent isotropic displacement parameters (Å^2^)

**Table:**

	*x*	*y*	*z*	*U*_iso_*/*U*_eq_	
O1	0.4567 (3)	0.50001 (9)	0.2429 (3)	0.0798 (8)	
O2	−0.0517 (2)	0.22010 (11)	0.2356 (2)	0.0676 (7)	
N1	0.7169 (3)	0.47041 (9)	0.3628 (3)	0.0533 (6)	
N2	0.7751 (3)	0.38557 (8)	0.2028 (3)	0.0442 (5)	
N3	0.2708 (3)	0.35681 (10)	0.1081 (3)	0.0594 (6)	
N4	0.2245 (2)	0.21276 (8)	0.3724 (2)	0.0389 (4)	
N5	0.3779 (2)	0.14267 (8)	0.2472 (2)	0.0387 (4)	
N6	−0.0202 (3)	0.18176 (10)	−0.1543 (2)	0.0542 (6)	
C1	0.7741 (4)	0.52148 (12)	0.4267 (4)	0.0659 (9)	
H1A	0.7646	0.5244	0.5200	0.079*	
H1B	0.7023	0.5469	0.3587	0.079*	
C2	0.9491 (4)	0.53244 (11)	0.4550 (3)	0.0554 (7)	
C3	1.0522 (6)	0.55963 (16)	0.5788 (5)	0.0892 (13)	
H3	1.0140	0.5697	0.6480	0.107*	
C4	1.2105 (7)	0.5723 (2)	0.6026 (7)	0.120 (2)	
H4	1.2771	0.5908	0.6871	0.144*	
C5	1.2692 (6)	0.5583 (2)	0.5057 (7)	0.1061 (17)	
H5	1.3760	0.5672	0.5222	0.127*	
C6	1.1719 (5)	0.53067 (18)	0.3822 (5)	0.0850 (11)	
H6	1.2128	0.5204	0.3150	0.102*	
C7	1.0129 (4)	0.51803 (13)	0.3574 (4)	0.0646 (8)	
H7	0.9474	0.4994	0.2728	0.077*	
C8	0.8150 (3)	0.42770 (10)	0.4385 (3)	0.0462 (6)	
C9	0.8904 (4)	0.42662 (13)	0.5933 (4)	0.0609 (8)	
H9	0.8712	0.4529	0.6464	0.073*	
C10	0.9937 (4)	0.38716 (15)	0.6696 (4)	0.0653 (8)	
H10	1.0424	0.3867	0.7734	0.078*	
C11	1.0243 (4)	0.34859 (13)	0.5920 (4)	0.0584 (7)	
H11	1.0934	0.3219	0.6434	0.070*	
C12	0.9533 (3)	0.34936 (11)	0.4390 (3)	0.0475 (6)	
H12	0.9785	0.3238	0.3874	0.057*	
C13	0.8435 (3)	0.38794 (9)	0.3592 (3)	0.0404 (5)	
C14	0.6213 (3)	0.39644 (10)	0.1254 (3)	0.0426 (6)	
C15	0.5592 (3)	0.39494 (11)	−0.0400 (3)	0.0507 (7)	
C16	0.6396 (4)	0.36447 (14)	−0.1030 (4)	0.0643 (8)	
H16	0.7246	0.3430	−0.0427	0.077*	
C17	0.5928 (6)	0.36622 (19)	−0.2549 (5)	0.0898 (14)	
H17	0.6471	0.3461	−0.2967	0.108*	
C18	0.4649 (7)	0.3979 (2)	−0.3453 (5)	0.0981 (16)	
H18	0.4348	0.3993	−0.4474	0.118*	
C19	0.3842 (6)	0.4266 (2)	−0.2859 (5)	0.0903 (14)	
H19	0.2975	0.4474	−0.3473	0.108*	
C20	0.4305 (4)	0.42525 (15)	−0.1321 (4)	0.0696 (9)	
H20	0.3739	0.4450	−0.0918	0.084*	
C21	0.5079 (3)	0.41522 (11)	0.1880 (3)	0.0473 (6)	
C22	0.5557 (3)	0.46494 (11)	0.2668 (4)	0.0531 (7)	
C23	0.3567 (3)	0.39855 (12)	0.1694 (3)	0.0522 (7)	
H23	0.3030	0.4207	0.2069	0.063*	
C24	0.1156 (4)	0.34505 (16)	0.1107 (5)	0.0756 (10)	
H24A	0.0819	0.3731	0.1530	0.113*	
H24B	0.1279	0.3152	0.1703	0.113*	
H24C	0.0335	0.3390	0.0107	0.113*	
C25	0.3329 (4)	0.31667 (13)	0.0448 (4)	0.0618 (8)	
H25A	0.4515	0.3170	0.0918	0.093*	
H25B	0.2938	0.3222	−0.0606	0.093*	
H25C	0.2941	0.2843	0.0614	0.093*	
C26	0.2094 (3)	0.22775 (11)	0.5084 (3)	0.0433 (6)	
H26A	0.1039	0.2160	0.5017	0.052*	
H26B	0.2108	0.2645	0.5151	0.052*	
C27	0.3454 (3)	0.20659 (10)	0.6471 (3)	0.0385 (5)	
C28	0.4192 (4)	0.23647 (11)	0.7735 (3)	0.0506 (7)	
H28	0.3895	0.2705	0.7694	0.061*	
C29	0.5365 (4)	0.21660 (14)	0.9058 (3)	0.0643 (8)	
H29	0.5844	0.2371	0.9900	0.077*	
C30	0.5822 (4)	0.16642 (15)	0.9128 (3)	0.0644 (8)	
H30	0.6591	0.1527	1.0024	0.077*	
C31	0.5143 (4)	0.13667 (13)	0.7877 (4)	0.0630 (8)	
H31	0.5475	0.1030	0.7916	0.076*	
C32	0.3955 (4)	0.15661 (10)	0.6542 (3)	0.0507 (6)	
H32	0.3498	0.1362	0.5696	0.061*	
C33	0.3757 (3)	0.22508 (9)	0.3639 (2)	0.0344 (5)	
C34	0.4547 (3)	0.27062 (10)	0.4213 (3)	0.0414 (5)	
H34	0.4093	0.2930	0.4659	0.050*	
C35	0.6004 (3)	0.28346 (11)	0.4134 (3)	0.0470 (6)	
H35	0.6518	0.3143	0.4514	0.056*	
C36	0.6686 (3)	0.24983 (11)	0.3485 (3)	0.0462 (6)	
H36	0.7653	0.2584	0.3410	0.055*	
C37	0.5936 (3)	0.20385 (11)	0.2952 (3)	0.0414 (5)	
H37	0.6430	0.1810	0.2555	0.050*	
C38	0.4454 (3)	0.19078 (9)	0.2991 (2)	0.0346 (5)	
C39	0.2219 (3)	0.13862 (9)	0.1633 (2)	0.0347 (5)	
C40	0.1538 (3)	0.08647 (10)	0.1183 (3)	0.0399 (5)	
C41	0.2590 (4)	0.04589 (12)	0.1369 (4)	0.0618 (8)	
H41	0.3723	0.0513	0.1747	0.074*	
C42	0.1952 (5)	−0.00297 (13)	0.0990 (5)	0.0770 (10)	
H42	0.2665	−0.0302	0.1126	0.092*	
C43	0.0313 (5)	−0.01128 (13)	0.0430 (4)	0.0743 (10)	
H43	−0.0097	−0.0441	0.0168	0.089*	
C44	−0.0745 (4)	0.02783 (13)	0.0242 (4)	0.0669 (9)	
H44	−0.1875	0.0218	−0.0140	0.080*	
C45	−0.0137 (4)	0.07677 (11)	0.0622 (3)	0.0526 (7)	
H45	−0.0866	0.1034	0.0498	0.063*	
C46	0.1031 (3)	0.18180 (9)	0.1194 (3)	0.0362 (5)	
C47	0.0810 (3)	0.20655 (10)	0.2424 (3)	0.0396 (5)	
C48	−0.0072 (3)	0.19553 (10)	−0.0206 (3)	0.0424 (6)	
H48	−0.0873	0.2185	−0.0233	0.051*	
C49	−0.1540 (5)	0.2023 (2)	−0.2892 (4)	0.0953 (14)	
H49A	−0.2265	0.2222	−0.2622	0.143*	
H49B	−0.2149	0.1749	−0.3529	0.143*	
H49C	−0.1084	0.2233	−0.3411	0.143*	
C50	0.0990 (4)	0.15021 (15)	−0.1764 (4)	0.0656 (9)	
H50A	0.2077	0.1573	−0.0999	0.098*	
H50B	0.0957	0.1573	−0.2725	0.098*	
H50C	0.0729	0.1151	−0.1715	0.098*	

### Atomic displacement parameters (Å^2^)

**Table:**

	*U*^11^	*U*^22^	*U*^33^	*U*^12^	*U*^13^	*U*^23^
O1	0.0545 (13)	0.0591 (13)	0.116 (2)	0.0199 (11)	0.0279 (13)	−0.0034 (14)
O2	0.0329 (9)	0.1096 (18)	0.0546 (11)	0.0214 (10)	0.0135 (8)	−0.0147 (12)
N1	0.0455 (12)	0.0397 (11)	0.0682 (15)	0.0073 (10)	0.0186 (11)	−0.0038 (11)
N2	0.0361 (11)	0.0416 (11)	0.0536 (13)	−0.0007 (9)	0.0180 (10)	−0.0034 (10)
N3	0.0413 (13)	0.0622 (15)	0.0681 (16)	−0.0048 (11)	0.0175 (12)	0.0114 (13)
N4	0.0310 (9)	0.0552 (12)	0.0319 (9)	0.0041 (9)	0.0151 (8)	−0.0025 (9)
N5	0.0324 (10)	0.0427 (10)	0.0393 (10)	0.0059 (8)	0.0139 (9)	−0.0010 (9)
N6	0.0511 (13)	0.0698 (15)	0.0336 (10)	0.0000 (12)	0.0110 (10)	0.0079 (11)
C1	0.070 (2)	0.0432 (15)	0.085 (2)	0.0054 (14)	0.0341 (18)	−0.0158 (15)
C2	0.0656 (19)	0.0390 (14)	0.0534 (16)	−0.0019 (12)	0.0180 (14)	−0.0040 (12)
C3	0.108 (3)	0.079 (3)	0.076 (3)	−0.021 (2)	0.035 (2)	−0.028 (2)
C4	0.114 (4)	0.105 (4)	0.101 (4)	−0.055 (3)	0.010 (3)	−0.028 (3)
C5	0.079 (3)	0.099 (3)	0.115 (4)	−0.029 (3)	0.019 (3)	0.022 (3)
C6	0.072 (2)	0.095 (3)	0.086 (3)	0.000 (2)	0.033 (2)	0.020 (2)
C7	0.066 (2)	0.0657 (19)	0.0523 (17)	−0.0016 (16)	0.0167 (15)	−0.0047 (15)
C8	0.0367 (13)	0.0426 (13)	0.0571 (16)	0.0019 (10)	0.0184 (12)	−0.0028 (12)
C9	0.0576 (18)	0.0663 (19)	0.0562 (17)	0.0075 (15)	0.0220 (15)	−0.0102 (15)
C10	0.0563 (18)	0.081 (2)	0.0472 (16)	0.0087 (17)	0.0115 (14)	0.0015 (16)
C11	0.0450 (15)	0.0605 (17)	0.0595 (18)	0.0118 (14)	0.0131 (14)	0.0101 (15)
C12	0.0353 (13)	0.0443 (13)	0.0607 (17)	0.0044 (11)	0.0186 (12)	−0.0018 (12)
C13	0.0296 (11)	0.0415 (12)	0.0510 (14)	−0.0006 (10)	0.0183 (11)	0.0001 (11)
C14	0.0328 (12)	0.0395 (12)	0.0543 (15)	−0.0053 (10)	0.0176 (11)	0.0002 (11)
C15	0.0436 (14)	0.0525 (15)	0.0509 (15)	−0.0154 (12)	0.0156 (12)	−0.0009 (13)
C16	0.0627 (19)	0.0666 (19)	0.0628 (19)	−0.0213 (16)	0.0264 (16)	−0.0170 (16)
C17	0.106 (3)	0.101 (3)	0.068 (2)	−0.039 (3)	0.043 (3)	−0.030 (2)
C18	0.106 (4)	0.124 (4)	0.053 (2)	−0.048 (3)	0.024 (2)	−0.006 (2)
C19	0.085 (3)	0.102 (3)	0.063 (2)	−0.022 (2)	0.011 (2)	0.019 (2)
C20	0.0540 (18)	0.082 (2)	0.0628 (19)	−0.0079 (17)	0.0154 (15)	0.0126 (18)
C21	0.0332 (13)	0.0510 (15)	0.0534 (15)	0.0041 (11)	0.0148 (12)	0.0076 (12)
C22	0.0406 (14)	0.0514 (15)	0.0709 (19)	0.0110 (12)	0.0276 (14)	0.0037 (14)
C23	0.0382 (14)	0.0561 (17)	0.0598 (17)	0.0065 (12)	0.0188 (13)	0.0109 (14)
C24	0.0465 (17)	0.080 (2)	0.103 (3)	−0.0055 (16)	0.0344 (18)	0.020 (2)
C25	0.0614 (19)	0.0594 (18)	0.0644 (19)	−0.0027 (15)	0.0271 (16)	0.0008 (15)
C26	0.0387 (13)	0.0580 (15)	0.0384 (12)	0.0078 (11)	0.0215 (11)	−0.0030 (11)
C27	0.0376 (12)	0.0487 (13)	0.0354 (11)	0.0012 (10)	0.0215 (10)	0.0004 (10)
C28	0.0554 (16)	0.0528 (16)	0.0432 (14)	0.0052 (12)	0.0209 (12)	−0.0048 (12)
C29	0.0660 (19)	0.079 (2)	0.0407 (14)	0.0038 (17)	0.0168 (14)	−0.0119 (15)
C30	0.0549 (18)	0.085 (2)	0.0454 (16)	0.0136 (16)	0.0146 (14)	0.0119 (16)
C31	0.069 (2)	0.0517 (16)	0.0672 (19)	0.0133 (15)	0.0283 (17)	0.0141 (15)
C32	0.0550 (16)	0.0454 (14)	0.0479 (14)	−0.0003 (12)	0.0189 (13)	−0.0027 (12)
C33	0.0278 (10)	0.0450 (12)	0.0286 (10)	0.0033 (9)	0.0104 (9)	0.0007 (9)
C34	0.0423 (13)	0.0458 (14)	0.0370 (12)	0.0023 (10)	0.0180 (11)	−0.0049 (10)
C35	0.0473 (15)	0.0484 (14)	0.0426 (13)	−0.0073 (11)	0.0168 (12)	−0.0031 (11)
C36	0.0342 (13)	0.0617 (16)	0.0437 (14)	−0.0055 (11)	0.0177 (11)	0.0025 (12)
C37	0.0344 (12)	0.0540 (14)	0.0371 (12)	0.0058 (11)	0.0165 (10)	−0.0002 (11)
C38	0.0299 (11)	0.0410 (12)	0.0286 (10)	0.0046 (9)	0.0086 (9)	0.0013 (9)
C39	0.0341 (11)	0.0401 (12)	0.0307 (10)	0.0051 (9)	0.0146 (9)	−0.0009 (9)
C40	0.0404 (13)	0.0433 (13)	0.0334 (11)	0.0027 (10)	0.0134 (10)	0.0000 (10)
C41	0.0542 (17)	0.0476 (15)	0.073 (2)	0.0075 (14)	0.0173 (15)	−0.0092 (15)
C42	0.082 (3)	0.0462 (17)	0.091 (3)	0.0060 (17)	0.026 (2)	−0.0073 (17)
C43	0.091 (3)	0.0453 (17)	0.068 (2)	−0.0155 (17)	0.018 (2)	−0.0056 (15)
C44	0.0591 (19)	0.066 (2)	0.0635 (19)	−0.0203 (16)	0.0147 (16)	0.0001 (16)
C45	0.0459 (15)	0.0496 (15)	0.0548 (16)	−0.0038 (12)	0.0147 (13)	0.0027 (13)
C46	0.0301 (11)	0.0399 (12)	0.0341 (11)	0.0030 (9)	0.0096 (9)	0.0003 (10)
C47	0.0322 (12)	0.0471 (13)	0.0392 (12)	0.0082 (10)	0.0150 (10)	0.0020 (11)
C48	0.0351 (12)	0.0457 (13)	0.0428 (13)	0.0030 (10)	0.0135 (10)	0.0047 (11)
C49	0.078 (2)	0.147 (4)	0.0382 (16)	0.010 (3)	0.0034 (16)	0.020 (2)
C50	0.075 (2)	0.084 (2)	0.0486 (16)	−0.0062 (18)	0.0373 (16)	−0.0038 (16)

### Geometric parameters (Å, °)

**Table:**

O1—C22	1.231 (3)	C21—C23	1.360 (4)
O2—C47	1.216 (3)	C21—C22	1.489 (4)
N1—C22	1.352 (4)	C23—H23	0.9300
N1—C8	1.425 (3)	C24—H24A	0.9600
N1—C1	1.480 (4)	C24—H24B	0.9600
N2—C14	1.288 (3)	C24—H24C	0.9600
N2—C13	1.403 (3)	C25—H25A	0.9600
N3—C23	1.328 (4)	C25—H25B	0.9600
N3—C24	1.438 (4)	C25—H25C	0.9600
N3—C25	1.458 (5)	C26—C27	1.497 (4)
N4—C47	1.383 (3)	C26—H26A	0.9700
N4—C33	1.432 (3)	C26—H26B	0.9700
N4—C26	1.469 (3)	C27—C28	1.383 (4)
N5—C39	1.286 (3)	C27—C32	1.382 (4)
N5—C38	1.402 (3)	C28—C29	1.382 (4)
N6—C48	1.332 (4)	C28—H28	0.9300
N6—C50	1.444 (4)	C29—C30	1.375 (5)
N6—C49	1.461 (4)	C29—H29	0.9300
C1—C2	1.499 (5)	C30—C31	1.369 (5)
C1—H1A	0.9700	C30—H30	0.9300
C1—H1B	0.9700	C31—C32	1.395 (4)
C2—C7	1.375 (5)	C31—H31	0.9300
C2—C3	1.377 (5)	C32—H32	0.9300
C3—C4	1.376 (8)	C33—C34	1.383 (3)
C3—H3	0.9300	C33—C38	1.403 (3)
C4—C5	1.333 (9)	C34—C35	1.385 (4)
C4—H4	0.9300	C34—H34	0.9300
C5—C6	1.367 (7)	C35—C36	1.384 (4)
C5—H5	0.9300	C35—H35	0.9300
C6—C7	1.379 (5)	C36—C37	1.374 (4)
C6—H6	0.9300	C36—H36	0.9300
C7—H7	0.9300	C37—C38	1.391 (3)
C8—C9	1.386 (4)	C37—H37	0.9300
C8—C13	1.398 (4)	C39—C46	1.488 (3)
C9—C10	1.379 (5)	C39—C40	1.490 (4)
C9—H9	0.9300	C40—C45	1.382 (4)
C10—C11	1.372 (5)	C40—C41	1.384 (4)
C10—H10	0.9300	C41—C42	1.391 (5)
C11—C12	1.371 (4)	C41—H41	0.9300
C11—H11	0.9300	C42—C43	1.346 (5)
C12—C13	1.397 (4)	C42—H42	0.9300
C12—H12	0.9300	C43—C44	1.357 (5)
C14—C21	1.488 (4)	C43—H43	0.9300
C14—C15	1.489 (4)	C44—C45	1.385 (4)
C15—C20	1.375 (5)	C44—H44	0.9300
C15—C16	1.394 (5)	C45—H45	0.9300
C16—C17	1.380 (5)	C46—C48	1.360 (3)
C16—H16	0.9300	C46—C47	1.472 (3)
C17—C18	1.388 (8)	C48—H48	0.9300
C17—H17	0.9300	C49—H49A	0.9600
C18—C19	1.346 (7)	C49—H49B	0.9600
C18—H18	0.9300	C49—H49C	0.9600
C19—C20	1.399 (6)	C50—H50A	0.9600
C19—H19	0.9300	C50—H50B	0.9600
C20—H20	0.9300	C50—H50C	0.9600
			
C22—N1—C8	121.1 (2)	H24B—C24—H24C	109.5
C22—N1—C1	117.8 (2)	N3—C25—H25A	109.5
C8—N1—C1	118.0 (2)	N3—C25—H25B	109.5
C14—N2—C13	119.6 (2)	H25A—C25—H25B	109.5
C23—N3—C24	123.0 (3)	N3—C25—H25C	109.5
C23—N3—C25	122.9 (3)	H25A—C25—H25C	109.5
C24—N3—C25	113.9 (3)	H25B—C25—H25C	109.5
C47—N4—C33	119.68 (19)	N4—C26—C27	112.6 (2)
C47—N4—C26	118.08 (19)	N4—C26—H26A	109.1
C33—N4—C26	117.39 (19)	C27—C26—H26A	109.1
C39—N5—C38	119.3 (2)	N4—C26—H26B	109.1
C48—N6—C50	123.8 (2)	C27—C26—H26B	109.1
C48—N6—C49	119.7 (3)	H26A—C26—H26B	107.8
C50—N6—C49	116.2 (3)	C28—C27—C32	118.5 (2)
N1—C1—C2	113.3 (2)	C28—C27—C26	120.2 (2)
N1—C1—H1A	108.9	C32—C27—C26	121.2 (2)
C2—C1—H1A	108.9	C27—C28—C29	121.1 (3)
N1—C1—H1B	108.9	C27—C28—H28	119.5
C2—C1—H1B	108.9	C29—C28—H28	119.5
H1A—C1—H1B	107.7	C30—C29—C28	119.9 (3)
C7—C2—C3	116.7 (4)	C30—C29—H29	120.0
C7—C2—C1	122.5 (3)	C28—C29—H29	120.0
C3—C2—C1	120.8 (4)	C31—C30—C29	119.8 (3)
C4—C3—C2	121.6 (5)	C31—C30—H30	120.1
C4—C3—H3	119.2	C29—C30—H30	120.1
C2—C3—H3	119.2	C30—C31—C32	120.3 (3)
C5—C4—C3	120.7 (5)	C30—C31—H31	119.8
C5—C4—H4	119.7	C32—C31—H31	119.8
C3—C4—H4	119.7	C27—C32—C31	120.3 (3)
C4—C5—C6	119.7 (5)	C27—C32—H32	119.8
C4—C5—H5	120.1	C31—C32—H32	119.8
C6—C5—H5	120.1	C34—C33—C38	119.7 (2)
C5—C6—C7	119.9 (5)	C34—C33—N4	120.1 (2)
C5—C6—H6	120.1	C38—C33—N4	120.2 (2)
C7—C6—H6	120.1	C33—C34—C35	121.1 (2)
C2—C7—C6	121.4 (4)	C33—C34—H34	119.5
C2—C7—H7	119.3	C35—C34—H34	119.5
C6—C7—H7	119.3	C36—C35—C34	119.3 (3)
C9—C8—C13	119.3 (2)	C36—C35—H35	120.4
C9—C8—N1	119.7 (3)	C34—C35—H35	120.4
C13—C8—N1	121.0 (3)	C37—C36—C35	120.1 (2)
C10—C9—C8	121.0 (3)	C37—C36—H36	120.0
C10—C9—H9	119.5	C35—C36—H36	120.0
C8—C9—H9	119.5	C36—C37—C38	121.4 (2)
C11—C10—C9	119.9 (3)	C36—C37—H37	119.3
C11—C10—H10	120.1	C38—C37—H37	119.3
C9—C10—H10	120.1	C37—C38—N5	118.6 (2)
C12—C11—C10	120.1 (3)	C37—C38—C33	118.4 (2)
C12—C11—H11	119.9	N5—C38—C33	122.8 (2)
C10—C11—H11	119.9	N5—C39—C46	124.5 (2)
C11—C12—C13	121.0 (3)	N5—C39—C40	117.5 (2)
C11—C12—H12	119.5	C46—C39—C40	117.8 (2)
C13—C12—H12	119.5	C45—C40—C41	118.1 (3)
C8—C13—C12	118.7 (2)	C45—C40—C39	121.6 (2)
C8—C13—N2	123.9 (2)	C41—C40—C39	120.2 (2)
C12—C13—N2	117.3 (2)	C40—C41—C42	120.0 (3)
N2—C14—C21	125.0 (2)	C40—C41—H41	120.0
N2—C14—C15	116.4 (2)	C42—C41—H41	120.0
C21—C14—C15	118.2 (2)	C43—C42—C41	120.7 (3)
C20—C15—C16	118.9 (3)	C43—C42—H42	119.7
C20—C15—C14	121.6 (3)	C41—C42—H42	119.7
C16—C15—C14	119.4 (3)	C42—C43—C44	120.5 (3)
C17—C16—C15	119.9 (4)	C42—C43—H43	119.7
C17—C16—H16	120.0	C44—C43—H43	119.7
C15—C16—H16	120.0	C43—C44—C45	119.9 (3)
C16—C17—C18	120.2 (4)	C43—C44—H44	120.1
C16—C17—H17	119.9	C45—C44—H44	120.1
C18—C17—H17	119.9	C40—C45—C44	120.8 (3)
C19—C18—C17	120.3 (4)	C40—C45—H45	119.6
C19—C18—H18	119.9	C44—C45—H45	119.6
C17—C18—H18	119.9	C48—C46—C47	115.5 (2)
C18—C19—C20	120.1 (4)	C48—C46—C39	127.9 (2)
C18—C19—H19	119.9	C47—C46—C39	115.57 (19)
C20—C19—H19	119.9	O2—C47—N4	120.7 (2)
C15—C20—C19	120.5 (4)	O2—C47—C46	124.4 (2)
C15—C20—H20	119.7	N4—C47—C46	114.92 (19)
C19—C20—H20	119.7	N6—C48—C46	131.1 (3)
C23—C21—C14	131.2 (3)	N6—C48—H48	114.5
C23—C21—C22	113.5 (3)	C46—C48—H48	114.5
C14—C21—C22	114.7 (2)	N6—C49—H49A	109.5
O1—C22—N1	121.2 (3)	N6—C49—H49B	109.5
O1—C22—C21	122.5 (3)	H49A—C49—H49B	109.5
N1—C22—C21	116.2 (2)	N6—C49—H49C	109.5
N3—C23—C21	132.5 (3)	H49A—C49—H49C	109.5
N3—C23—H23	113.7	H49B—C49—H49C	109.5
C21—C23—H23	113.7	N6—C50—H50A	109.5
N3—C24—H24A	109.5	N6—C50—H50B	109.5
N3—C24—H24B	109.5	H50A—C50—H50B	109.5
H24A—C24—H24B	109.5	N6—C50—H50C	109.5
N3—C24—H24C	109.5	H50A—C50—H50C	109.5
H24A—C24—H24C	109.5	H50B—C50—H50C	109.5
			
C22—N1—C1—C2	−147.1 (3)	C47—N4—C26—C27	148.3 (2)
C8—N1—C1—C2	52.7 (4)	C33—N4—C26—C27	−56.4 (3)
N1—C1—C2—C7	40.1 (5)	N4—C26—C27—C28	138.3 (3)
N1—C1—C2—C3	−142.6 (4)	N4—C26—C27—C32	−44.3 (3)
C7—C2—C3—C4	0.8 (6)	C32—C27—C28—C29	−2.2 (4)
C1—C2—C3—C4	−176.6 (4)	C26—C27—C28—C29	175.3 (3)
C2—C3—C4—C5	−0.3 (8)	C27—C28—C29—C30	0.5 (5)
C3—C4—C5—C6	−0.5 (9)	C28—C29—C30—C31	1.6 (5)
C4—C5—C6—C7	0.8 (8)	C29—C30—C31—C32	−1.8 (5)
C3—C2—C7—C6	−0.5 (5)	C28—C27—C32—C31	2.0 (4)
C1—C2—C7—C6	176.8 (3)	C26—C27—C32—C31	−175.5 (3)
C5—C6—C7—C2	−0.2 (6)	C30—C31—C32—C27	0.1 (5)
C22—N1—C8—C9	−121.1 (3)	C47—N4—C33—C34	117.1 (2)
C1—N1—C8—C9	38.4 (4)	C26—N4—C33—C34	−37.8 (3)
C22—N1—C8—C13	62.2 (4)	C47—N4—C33—C38	−63.7 (3)
C1—N1—C8—C13	−138.3 (3)	C26—N4—C33—C38	141.4 (2)
C13—C8—C9—C10	0.4 (5)	C38—C33—C34—C35	1.2 (4)
N1—C8—C9—C10	−176.3 (3)	N4—C33—C34—C35	−179.6 (2)
C8—C9—C10—C11	0.8 (5)	C33—C34—C35—C36	−0.7 (4)
C9—C10—C11—C12	0.4 (5)	C34—C35—C36—C37	−1.2 (4)
C10—C11—C12—C13	−2.8 (4)	C35—C36—C37—C38	2.7 (4)
C9—C8—C13—C12	−2.7 (4)	C36—C37—C38—N5	−178.1 (2)
N1—C8—C13—C12	174.0 (2)	C36—C37—C38—C33	−2.1 (3)
C9—C8—C13—N2	−179.2 (3)	C39—N5—C38—C37	−136.3 (2)
N1—C8—C13—N2	−2.6 (4)	C39—N5—C38—C33	48.0 (3)
C11—C12—C13—C8	3.9 (4)	C34—C33—C38—C37	0.2 (3)
C11—C12—C13—N2	−179.3 (3)	N4—C33—C38—C37	−179.0 (2)
C14—N2—C13—C8	−44.4 (4)	C34—C33—C38—N5	175.9 (2)
C14—N2—C13—C12	139.0 (2)	N4—C33—C38—N5	−3.3 (3)
C13—N2—C14—C21	4.4 (4)	C38—N5—C39—C46	−2.0 (3)
C13—N2—C14—C15	177.8 (2)	C38—N5—C39—C40	−176.3 (2)
N2—C14—C15—C20	−150.9 (3)	N5—C39—C40—C45	163.1 (2)
C21—C14—C15—C20	22.9 (4)	C46—C39—C40—C45	−11.5 (3)
N2—C14—C15—C16	25.1 (4)	N5—C39—C40—C41	−14.6 (4)
C21—C14—C15—C16	−161.1 (3)	C46—C39—C40—C41	170.7 (3)
C20—C15—C16—C17	1.9 (5)	C45—C40—C41—C42	0.2 (5)
C14—C15—C16—C17	−174.2 (3)	C39—C40—C41—C42	178.0 (3)
C15—C16—C17—C18	−0.6 (6)	C40—C41—C42—C43	0.6 (6)
C16—C17—C18—C19	−0.9 (7)	C41—C42—C43—C44	−0.9 (6)
C17—C18—C19—C20	1.0 (7)	C42—C43—C44—C45	0.4 (6)
C16—C15—C20—C19	−1.8 (5)	C41—C40—C45—C44	−0.7 (4)
C14—C15—C20—C19	174.2 (3)	C39—C40—C45—C44	−178.5 (3)
C18—C19—C20—C15	0.4 (6)	C43—C44—C45—C40	0.4 (5)
N2—C14—C21—C23	−128.0 (3)	N5—C39—C46—C48	127.8 (3)
C15—C14—C21—C23	58.7 (4)	C40—C39—C46—C48	−57.9 (3)
N2—C14—C21—C22	62.0 (4)	N5—C39—C46—C47	−64.5 (3)
C15—C14—C21—C22	−111.3 (3)	C40—C39—C46—C47	109.8 (2)
C8—N1—C22—O1	153.5 (3)	C33—N4—C47—O2	−144.3 (3)
C1—N1—C22—O1	−6.1 (5)	C26—N4—C47—O2	10.4 (4)
C8—N1—C22—C21	−29.0 (4)	C33—N4—C47—C46	37.1 (3)
C1—N1—C22—C21	171.4 (3)	C26—N4—C47—C46	−168.2 (2)
C23—C21—C22—O1	−39.9 (4)	C48—C46—C47—O2	30.9 (4)
C14—C21—C22—O1	131.8 (3)	C39—C46—C47—O2	−138.3 (3)
C23—C21—C22—N1	142.6 (3)	C48—C46—C47—N4	−150.5 (2)
C14—C21—C22—N1	−45.6 (4)	C39—C46—C47—N4	40.2 (3)
C24—N3—C23—C21	175.1 (3)	C50—N6—C48—C46	−5.8 (5)
C25—N3—C23—C21	1.0 (5)	C49—N6—C48—C46	179.4 (3)
C14—C21—C23—N3	10.9 (6)	C47—C46—C48—N6	178.1 (3)
C22—C21—C23—N3	−179.0 (3)	C39—C46—C48—N6	−14.2 (5)
